# Effects of Transcutaneous Electrical Nervous Stimulation (TENS) on Dysphonic Patients: A Systematic Review Study

**DOI:** 10.3390/medicina59101737

**Published:** 2023-09-28

**Authors:** Panagiotis Plotas, Angelos Papadopoulos, Eirini Tsiamaki, Maria-Dimitra Apostolou, Maria-Antonia Chaniotaki, Efthimia Ganiatsou, Eleni-Marianthi Goutzeri, Thalia Kalogeraki, Elpida Karra, Maria Malliou, Dimitra Marinitsi, Chariklia Papoutsaki, Ilianna-Stamatia Vagianou, Nikolaos Trimmis

**Affiliations:** 1 Laboratory of Primary Health Care, School of Health Rehabilitation Sciences, University of Patras, 26504 Patras, Greece; 2 Department of Speech and Language Therapy, School of Health Rehabilitation Sciences, University of Patras, 26504 Patras, Greece; up1076104@upnet.gr (M.-D.A.); mariachaniotaki98@gmail.com (M.-A.C.); p1080003@upnet.gr (E.G.); upw800128@upnet.gr (E.-M.G.); up1078386@upnet.gr (T.K.); up1076093@upnet.gr (E.K.); up1076095@upnet.gr (M.M.); dimitra.marinitsi@gmail.com (D.M.); up1076109@upnet.gr (C.P.); up1074145@upnet.gr (I.-S.V.); nicktrimmis@upatras.gr (N.T.); 3 General Children’s Hospital of Patras “Karamandaneio”, 26331 Patras, Greece; 4 Department of Neurology, School of Medicine, University of Patras, 26504 Patras, Greece; eirinitsiamaki@gmail.com

**Keywords:** TENS, systematic review, dysphonia, voice disorders, voice therapy

## Abstract

*Background and Objectives:* Transcutaneous electrical nerve stimulation (TENS), a pain-alleviating and muscle-relaxing treatment used in physio-therapeutic clinical practice, has recently appeared to be just as effective in dysphonia. This review aimed at clarifying whether TENS can be an effective practice in dysphonia therapy and/or management on its own or combined with other types of interventions and, hence, whether its practice can be a useful, more widespread establishment to speech and language therapy intervention methods. *Materials and Methods:* A search was conducted on the PubMed database using specific terms based on the PICO search strategy. Eventually, four randomized controlled studies and four clinical trials were included. The methodological quality of the included studies was evaluated using the physiotherapy evidence-based database (PEDro) assessment tool, and this indicated high-quality research with an average score of 8.43. *Results:* The studies utilized various TENS devices, predominantly the Dualpex 961 device (frequency of 10 Hz, phase of 200 ms). The assessment methods varied, including auditory perception, vocal therapy, electrostimulation, audio and video perceptual assessments, and laryngeal evaluations. The clinical outcomes of TENS showed a reduction in musculoskeletal pain in various areas, while the acoustic analysis results were significant in only one study. TENS was compared to manual laryngeal therapy (LMT), placebo TENS, and vocal therapy in different studies with mixed results. *Conclusions:* This review supports the idea that a multidimensional approach, incorporating various therapeutic modalities (TENS, LMT, speech therapy, and vocal training) can yield positive outcomes for patients with voice disorders. Further research is needed to explore the specific mechanisms of action and optimal treatment protocols for TENS in voice therapy.

## 1. Background

In order to achieve vocal production, body systems such as breathing, phonation, articulation, and resonance must function and coordinate properly. Impairment of the muscle structures involved in the aforementioned processes can cause a negative impact on vocal quality. Dysphonia, the alteration in normal voice quality, can occur due to structural and/or functional reasons and is characterized by various symptoms and physical manifestations of vocal changes, such as throat clearing, soreness, neck tightness, breathiness, poor voice quality, and sudden vocal attacks, as well as excessive tension of the laryngeal extrinsic muscles [1,2]. Voice assessment is important, especially in clinical practice, to test voice capabilities, to quantify the [3] degree of dysphonia, and to assess the results of treatment [3]. Dysphonia can be treated using indirect and direct vocal therapy methods, including practices that promote healthy vocal habits and vocal techniques with proven efficacy [4]. Numerous voice therapy strategies have been suggested, including vocal function exercises (VFEs), resonant voice therapy (RVT), and manual circumlaryngeal therapy (MCT) [5,6]. Vocal function exercises (VFEs) encompass a set of methodical vocal manipulations aimed at enhancing the power and endurance of the muscles of the larynx [5,6]. These exercises also aid in the facilitation of laryngeal valving, control over phonation, and expansion of the vocal range. Resonant voice therapy (RVT) is a therapeutic approach that aims to assist individuals in attaining a targeted vocal fold configuration by educating them to perceive facial vibrations. This technique has demonstrated notable efficacy, particularly among individuals who rely on their voices professionally, such as teachers and singers [5,6]. The modified circumlaryngeal massage technique (MCT) is a frequently employed method in voice therapy that aims to alleviate laryngeal tension and mitigate the symptoms of dysphonia [5,6]. Additionally, techniques that focus on muscle relaxation, such as manual laryngeal therapy and transcutaneous electrical nerve stimulation (TENS), have been shown to be effective in speech therapy for individuals with dysphonic voice disorders [7].

TENS is a non-invasive treatment that has traditionally been used to alleviate pain and promote muscle relaxation in different medical settings, involving the use of a mild electrical current [8]. Recently, it has been introduced into speech therapy practice as a new important therapeutic strategy for voice functions. Depending on the different types of this method’s application, TENS can stimulate different physiological mechanisms [8]. Low-frequency TENS (≤10 Hz) interacts with the peripheral nervous system, generating muscle contractions, while high-frequency TENS (≥50 Hz) elicits paresthesia [8]. Both are used in the management of acute and chronic pain, causing analgesia. Many studies have been carried out in this clinical field, demonstrating the growing utilization of new resources in speech therapy and showing that the use of TENS can lead to positive outcomes regarding voice and swallowing disorders [8]. While TENS, regarding the rehabilitation of dysphonia, is useful in relaxing the cervical and perilaryngeal muscles and is considered a good therapeutic tool, it is most effective when used alongside conventional vocal therapy in order to further increase the voice quality [8].

It is of great importance to continue exploring new methods and strategies to be used in the SLP clinic area regarding muscular and vocal rehabilitation. Given the prevalence of dysphonia associated with muscle tension and the increasing use of new technologies in therapeutic methods, clinical research is the only way of developing effective tools to best treat patients. This systematic review aims to identify, evaluate, and summarize the application methods and clinical outcomes of TENS in the rehabilitation of dysphonic patients and to contribute towards the speech therapy field.

## 2. Materials and Methods

### 2.1. Study Design and Research Strategies

**Figure 1:**
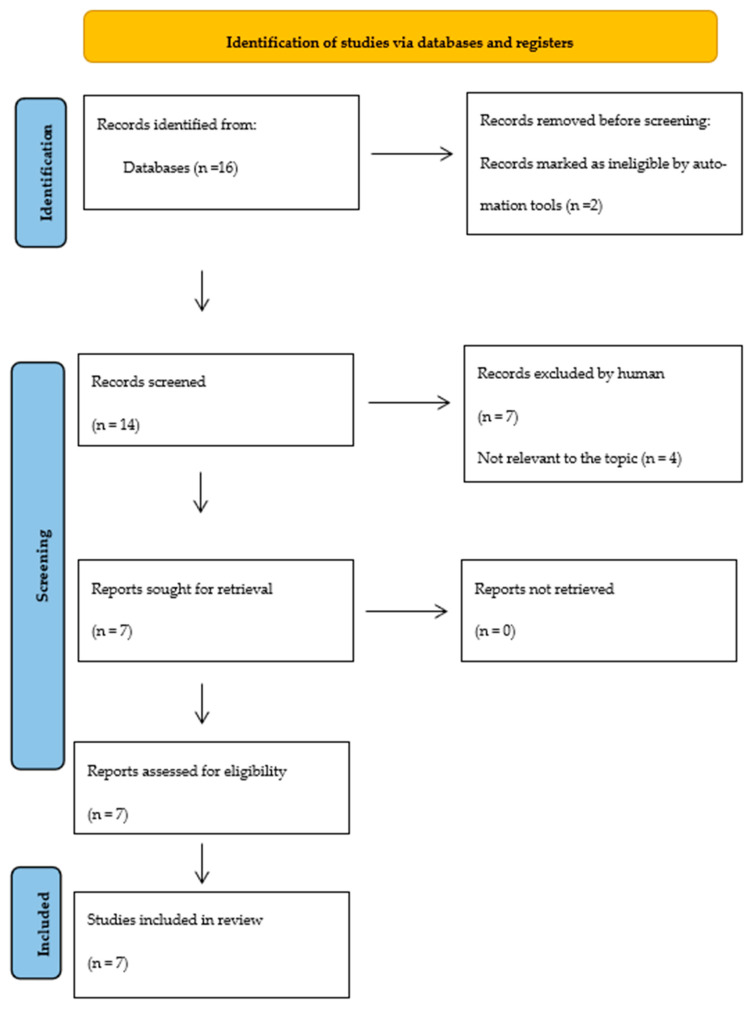
The PRISMA 2020 statement: an updated guideline for reporting systematic reviews.

As a systematic review study, the overall goal was to investigate and clarify the effectiveness of TENS (transcutaneous electric nerve stimulation) therapy in individuals with a dysphonia diagnosis. The search was conducted on 12 April 2023 on the PubMed database. The terms “TENS”, “dysphonia therapy”, and “effectiveness” were input into the searching machine based on the PICO search strategy [9,10,11] using the Boolean term “AND” for the PubMed builder’s comprehension. More details about the process are presented in Figure 1, “The PRISMA 2020 statement: an updated guideline for reporting systematic reviews” [12].

### 2.2. Eligibility Criteria and Data Extraction

The selection of the articles was implemented by methodically following some steps. We excluded articles whose titles were not associated with the designated terms, and their type was regarded as a systematic or meta-analysis review. The abstracts of the articles were read in detail, and, subsequently, we selected which studies with randomized controlled trials (RCT) and clinical trials were appropriate and described clearly the concept of the application methods of TENS and its clinical effects in patients with dysphonia that were published in the last 10 years in English. The exclusion criteria encompass the removal of any studies with neurological or oncological patients. The final and longest-lasting stage of the data extraction was reading each whole article carefully so as to select those which fulfilled the eligibility criteria. Moreover, the diagnostic criteria in all the studies analyzed in this review required a dysphonia confirmation through an otolaryngologist examination. This examination includes endoscopic voice evaluation. The following information was taken from the full-text reading: title, year, journal, primary author, study design, aim, sample characteristics, protocols and scales of voice assessment before and after the intervention used, and the clinical outcomes of intervention. More details about the studies and sample characterization in Table 1. 

### 2.3. Methodological Evaluation

The physiotherapy evidence-based database (PEDro) [13] is an assessment tool based on the Delphi scale developed by Verhagen and colleagues at the University of Maastricht, which can reliably define the methodological quality of clinical randomized controlled trials [14,15]. The scale includes 11 criteria, and each one is graded as either positive/present (1) or negative/absent (0). However, only 10 out of 11 items are graded with a maximum score of up to 10, providing that the first one of utmost importance (eligibility criteria) is positive about the ability of the inclusion and exclusion criteria in order to make the results of the experimental studies generalizable to a specific population. Items 2 through 11 are about the internal validity, statistics, and outcome measures of the study. Informatively, a clinical trial is regarded as moderate-to-high quality when the total score is at least 6 out of 10 [16]. In the current systematic review, the evaluation of the methodological quality of the studies was carried out by ten independent researchers.

**Table 1:** Sample characterization.

Study	Title	Sample	Age	Gender	Study Type
[7]	Effects of laryngeal manual therapy (LMT) and transcutaneous electrical nerve stimulation (TENS) in vocal folds diadochokinesis of dysphonic women: a randomized clinical trial	Women with bilateral vocal nodules participated	18–45	20 Female	Randomized clinical trial
[1]	Effect of Vocal Therapy Associated with TENS in Women with Behavioral Dysphonia	Women with behavioral dysphonia	Average 46.1 (±15.1) years	17 Female	Clinical trial
[17]	Effects of transcutaneous electrical nervous stimulation (TENS) associated with vocal therapy on musculoskeletal pain of women with behavioral dysphonia: A randomized, placebo-controlled double-blind clinical trial	Women with behavioral dysphonia	Average (ΕΚ) 29 and Average (ΚΓ) 31.6	27 Female	A randomized, placebo-controlled, double-blind clinical trial
[4]	Effect of Application of Transcutaneous Electrical Nerve Stimulation and Laryngeal Manual Therapy in Dysphonic Women: Clinical Trial	Women with bilateral vocal nodules participated	18–45	20 Female	Clinical trial
[2]	Evaluation of Electrostimulation Effect in Women with Vocal Nodules	Women with an otolaryngology diagnosis of vocal fold nodules using a videolaryngostroboscopy examination	18–55	60 Female	Randomized, prospective, comparative, intra-subject study
[18]	Transcutaneous Electrical Nerve Stimulation (TENS) and Laryngeal Manual Therapy (LMT): Immediate Effects in Women with Dysphonia	Women with behavioral dysphonia	18–45	30 Female	Clinical trial
[8]	Application of High-Frequency Transcutaneous Electrical Nerve Stimulation in Muscle Tension Dysphonia Patients with the Pain Complaint: The Immediate Effect	Patients with muscle tension dysphonia (MTD)	Average 36.40 (±5.76 years)	20 Female 10 male	Clinical trial

## 3. Results

### 3.1. Application Methods of TENS

In a 20 min session, participants were placed in a supine and comfortable position on a stretcher and asked to remain silent without vocalizations. Before the electrodes were placed, the skin was cleaned with alcohol gel, and the electrodes were secured with anti- allergic tape. For the application of TENS, most studies used a Dualpex 961 device as their equipment [4,7,17,18], while one used an ELPHA II 3000 device [8] and another used a NEURODYN II device [2]. All studies used a frequency of 10 Hz and a phase of 200 ms, except for the study in [8], which used high-frequency TENS (Table 2). The studies had parameters using symmetric, biphasic, square pulses, while two studies [8,18] used symmetric, biphasic, rectangular pulses. Electrodes were placed on the trapezius muscle (descending fiber) and on the submandibular region.

### 3.2. Assessment Methods

To verify the clinical outcomes of TENS, some parameters stood out for being frequently used in the randomized clinical trials. All seven of these studies used acoustic voice analysis with different programs. To be more specific, four studies used acoustic voice analysis with jitter and shimmer. The other two studies used the Sony Forge Pro 10 software and Sound Forge 10 for vocal quality. Specifically, the study that used Sound Forge 10 [7] used laryngeal diadochokinesis (DDK), in which the participant had to repeat the vowels /a/ and /i/ separately. Furthermore, two studies conducted NMSQ to carry out a pain survey in the form of a pain interview. Also, three surveys used the GRBAS scale for auditory perception. The other two studies used different scales. One of them [8] used the VTD scale and the other one [7] used the motor speech profile advanced (MSP) survey for vowel analysis. Finally, one study [17] used the pressure pain threshold (PPT), which was a musculoskeletal pain questionnaire.

### 3.3. Outcome Measures

**Figure 2:**
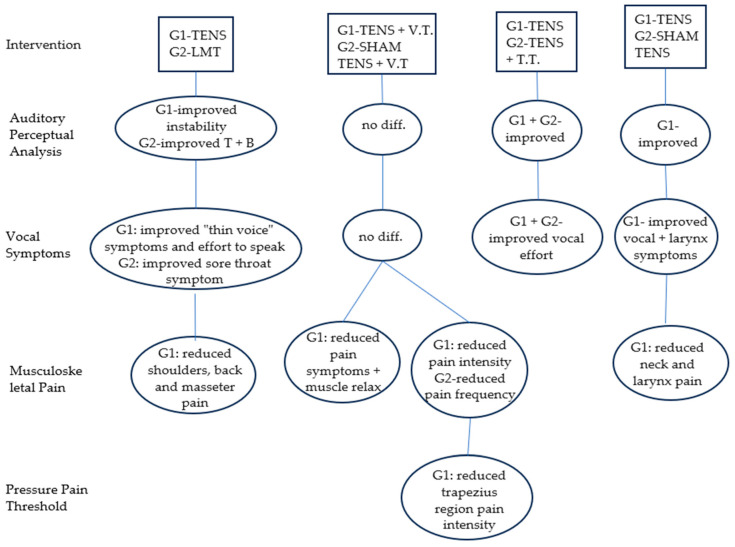
Schematic diagram of the effects of TENS application. B: breathiness, T: tension, T.T.: tongue trills, V.T.: voice therapy.

Figure 2 shows the interventions performed and the frequency and results of the interventions, and it compares the use of TENS with other treatment methods such as LMT, vocal therapy, and placebo TENS. Of the studies analyzed, 85.7% showed effective results after TENS application. The interventions were divided into twelve sessions (42.8%), six sessions (14.3%), or one session (42.8%). The studies [1,4,8,17,18] that assessed individuals’ musculoskeletal pain after TENS application showed a reduction in muscle pain, helping muscles to relax [1] in the posterior and anterior neck [2,8,18], shoulders [4,18], upper and lower back [8,18], temporal region [8,18], larynx [8,17], submandibular [8], and masseter [8,18]. Only one study showed significant results in the acoustic analysis with an improvement in the coordination of vocal fold movements during phonation [4]. In the perceptual analysis, the studies found improvements in the parameters of tension, breathiness, hoarseness, and instability [1,4]. The other studies did not present significant differences between groups. Three studies [4,7,18] compared the effects of TENS with the effects of manual laryngeal therapy (LMT) and obtained different results for each technique. In one study [7], LMT provided a greater regularity of movement during laryngeal diadochokinesis. In another study [4], after comparing the two techniques in relation to vocal/laryngeal symptoms, pain, and vocal quality, there was an improvement in the “sore throat”, a significantly lower prevalence of pain in the anterior neck, and the pain intensity in the posterior neck was reduced. In a third study [18], after LMT, a drop in pain intensity in the neck anterior region, shoulders, lower back, and temporal region was observed.

Moreover, two studies [1,18] analyzed the effects of placebo TENS combined with vocal therapy and did not present significant differences between groups. Moreover, a study [1] showed an improvement in vocal instability, vocal tension, and spontaneous speech for the hoarseness, breathiness, and tension. In addition, a study [17] indicated a decrease in the frequency and intensity of musculoskeletal pain in regions proximal to the larynx.

### 3.4. Quality of Studies

The methodological quality of the articles was analyzed through the PEDro scale [13], as seen in Table 3, and their mean score was 8.43. All seven studies were considered to be high-quality, with a score of 8 or above. In most of the studies, the groups were similar or they had no statistical differences, as determined by the paired *t*-test. The age range of the participants was from 18 to 60 years, and the intergroup comparison results were described. All the studies presented both precision and variability measures for at least one key result. Randomization was described in three studies [2,7,17], while the others were clinical trials with no randomization [1,4,8,18]. Criterion 9 was not scored in any of the studies, as no specific information was provided.

## 4. Discussion

In this review, the use and results of TENS in patients with dysphonia were examined. Regarding the clinical results, after the interventions with TENS, there were changes in vocal quality and an improvement in qualitative indicators.

A total of 204 people took part in the survey, with women making up the vast majority (194) and with only 10 men. Except for one article that did not specify the average age of its particular sample, the participants had an average age of 34. Similar studies have found that women have voice issues more frequently than men do. This might be explained by a number of elements, including societal conventions, obligations at work, and cultural expectations. Women frequently have to make more outspoken demands, maybe as a result of societal expectations and traditions put on them. Additionally, the diversity in vocal quality and pitch between males and females is influenced by physical differences. Generally speaking, women’s vocal folds are shorter than men’s, which might result in variations in voice qualities. Additionally, the vocal folds may experience increased stress as a result of social pressure for women to have higher-pitched voices, which could result in voice-related problems [19,20].

Studies have consistently shown a connection between vocal issues and musculoskeletal pain [21,22,23]. This discomfort, which is brought on by strains in the larynx and neck, is typically caused by poor vocal practices. One solution for this issue is transcutaneous electrical nerve stimulation (TENS). TENS is primarily used to relax the larynx, lessen pain, and enhance vocal quality in people with voice problems [1,7,8,17,18]. The findings of every study conducted on this subject indicated that using TENS significantly reduced pain symptoms. The areas of the upper back, shoulders, and the front and back of the neck in particular exhibited the highest improvement following this treatment.

The most frequent diagnosis observed in the sample was vocal nodules [1,2,7,17]. Vocal nodules are linked to repetitive lesions in the mucosa of the vocal folds, resulting in abuse of the voice and vocalization with hyperfunction of the larynx [24,25]. Vocal nodules are also observed more frequently in women [26].

A discernible pattern emerged from the research analyzed in this review concerning the application parameters of TENS. The majority of the investigations utilized low-frequency TENS with a frequency of 10 Hz and 200 pulses per minute [27]. The intensity of the stimulation varied based on the individual’s sensitivity. Specifically, when a patient reported diminished sensation, the stimulation was increased accordingly. Previous research has indicated that women are more prone to experiencing trigger points on the trapezius muscle due to persistent strain [18]. The placement of the electrodes plays a significant role in determining the region of stimulation, thereby yielding various effects [28]. By positioning one electrode from each channel strategically, an electrostimulation field is created, activating all perilaryngeal muscles. Consequently, the arrangement of the electric current generated by both channels induces electrostimulation of the muscle groups, providing a sense of calm to the larynx and trapezius muscle [28]. However, it should be noted that this approach also generates strong vibrations within these muscles. Ultimately, the reviewed studies produced consistent findings in support of these observations.

The studies analyzed in this review utilized different methodologies to assess the severity of musculoskeletal pain. For instance, one study [22] relied on the musculoskeletal pain investigation questionnaire [29] as a measurement tool, while another study [17] employed the pressure pain threshold test [30]. Furthermore, two additional studies [4,8] utilized the NMSQ-E scale [31] to evaluate levels of musculoskeletal pain. It is worth noting that there was heterogeneity in how pain and symptoms were evaluated across the reviewed articles, and the outcomes were interpreted in a broader context considering the varying scales used. The groups that underwent transcutaneous electrical nerve stimulation (TENS) exhibited improved pain tolerance and a reduction in pain symptoms in the respective areas. This scientific observation can be explained by the muscle contractions induced by TENS stimulation, which promote muscle relaxation and alleviate pain [27]. Interestingly, a noteworthy finding was the observed decrease in pain experienced in regions that were not directly stimulated [1].

The studies in [2,4,8,17,18] also indicate developments in the perceptual analysis’s metrics for roughness, breathiness, tension, and instability. We are aware that the clinical picture of muscle tension dysphonia MTD includes roughness [32,33], which is consistent with the findings of our study. The present study showed a sizable proportion of participants with rough voice quality. The roughness parameter saw a beneficial influence in some of the examined experiments after TENS operations. The vibrations produced in the larynx by electrical stimulation, which encourage harmony in the extrinsic and intrinsic muscles of this organ and result in appropriate closure of the vocal folds, might be the cause of the progress in the analysis of these parameters. According to neurological cases, asthenia, or vocal hypofunction, is associated with a sense of vocal fragility and a lack of strength in the emission [34].

The participants of the study in [18] reported a decrease in symptoms related to sore throat and discomfort while speaking, which can be attributed to the analgesic action, muscle relaxation, and enhancement in vocal quality brought about by TENS. These beneficial impacts are believed to stem from the restoration of muscular balance, which subsequently mitigates the symptoms caused by muscle contraction in the relevant areas [18].

The quality of the studies was evaluated using the PEDro scale [13]. Various voice therapy procedures have been proposed in the current literature (e.g., vocal function exercises, resonant voice therapy, and manual circumlaryngeal therapy) [5,6]. In addition, to improve the evidence regarding the effectiveness of the technique used, it is recommended that further research is needed. Moreover, given the limited number of studies evaluated in this research review, it is necessary to replicate the study with a greater number of future studies to strengthen the findings. These future studies should incorporate larger samples, be applied to diverse populations, and involve therapists and double-blinding techniques to more effectively verify the effects of TENS on aspects related to the voice. The studies examined in this review exhibited comparable outcomes in terms of the application procedures and clinical effects of TENS. This suggests that there are developing guidelines for implementing this technique in the treatment of voice disorders, and it holds potential as an adjunctive therapeutic approach for individuals with muscle tension dysphonia, producing positive effects.

## 5. Conclusions

In this study, it was found that vocal therapy, in combination with TENS, effectively reduced the frequency and intensity of musculoskeletal pain in regions near the larynx in women with vocal nodules. The threshold of sensitivity to muscle pain in the trapezius muscle was increased, indicating benefits from TENS therapy. However, both TENS and vocal therapy were found to be equally effective in treating behavioral dysphonia in women. Additionally, manual laryngeal therapy (LMT) was found to improve the regularity of diadochokinetic movements of the vocal folds, indicating its positive impact on phonatory function. On the other hand, TENS did not show any modifications in the laryngeal parameters of the studies. Electrical stimulation combined with vocal therapy reduced the electrical activity of the infrahyoid muscles and improved the temperature balance between the supra- and infrahyoid regions in women with behavioral dysphonia. Overall, this study suggests that TENS and LMT are valuable therapeutic approaches, but when used in conjunction with speech therapy and vocal training, they can further enhance voice quality and laryngeal aspects. The findings of this study indicate that both TENS and vocal therapy, either used alone or in combination, can be effective in treating musculoskeletal pain and certain vocal symptoms in individuals with dysphonia.

## 6. Future Directions

The results support the idea that a multidimensional approach, incorporating various therapeutic modalities, including TENS, LMT, speech therapy, and vocal training, can yield positive outcomes for patients with voice disorders. However, it is important to consider individual patient characteristics and tailor the treatment approach accordingly. Further research is needed to explore the specific mechanisms of action and optimal treatment protocols for TENS in voice therapy.

## Figures and Tables

**Table 2:** TENS application characteristics.

Study	Device	Frequency	Pulse Width	Time	Location of the Electrodes	Types of Electrodes
[7]	Dualpex 961	10 Hz	200 μs	20 min	Trapezius muscle region (descending fibers) and submandibular region, bilaterally	2 pairs (3.0 cm × 4.0 cm)
[1]	Ibramed Neurodyn III Amethyst Line	15 Hz	250 μs	30 min	On clean skin, positioned on the upper border, at the level of the hyoid bone, and on the lower border, below the cricoid cartilage, one centimeter apart from one another	1 pair (3 cm × 5 cm)
[17]	Quark Dualpex 961	10 Hz	200 μs	20 min	Trapezius muscle and submandibular area	2 pairs (3 cm × 5 cm)
[4]	Dualpex 961	10 Hz	200 μs	20 min	Trapezius region—upper fibers, bilaterally, and in the submandibular region	2 pairs (5.0 cm × 5.0 cm)
[2]	NEURODYN II	10 Hz	200 μs	20 min	Lateral center of the larynx (thyroid cartilage) in the infrahyoid muscles, with 1 cm between them and on trapezius muscles	Rectangular and composed of silicon (30 × 50 mm)
[18]	Dualpex 961	10 Hz	200 μs	20 min	Trapezius muscle (on the descending fiber) and on both sides of the submandibular region	2 pairs (5.0 cm × 4.0 cm)
[8]	ELPHA II 3000	100 Hz	50 μs	20 min	Lateral center of the thyroid cartilage in the infrahyoid muscles and the motor point of the trapezius muscle on the descending fiber	2 pairs (5 cm × 5 cm)

**Table 3:** Methodological quality assessment.

	[14]	[1]	[15]	[4]	[2]	[16]	[5]
1. Eligibility criteria	YES	YES	YES	YES	YES	YES	YES
2. Randomized allocation	YES	YES	YES	YES	YES	YES	YES
3. Secret allocation	YES	YES	YES	YES	YES	NO	YES
4. Similarity of groups in prognosis	YES	YES	YES	YES	YES	YES	YES
5. Blinding of all subjects	YES	NO	NO	YES	YES	YES	YES
6. Blinding of all therapists	YES	YES	YES	YES	YES	YES	YES
7. Blinding of assessors	YES	YES	YES	YES	YES	YES	YES
8. Measures of at least one key outcome were obtained from more than 85% of the subjects initially allocated to groups	NO	NO	YES	YES	YES	YES	YES
9. Analysis of “intention to treat”	NO	NO	NO	NO	NO	NO	NO
10. Between-group statistical comparisons	YES	YES	YES	YES	YES	YES	YES
11. Precision and variability measures	YES	YES	YES	YES	YES	YES	YES
Total Points	8/10	8/10	8/10	9/10	9/10	8/10	9/10

## Data Availability

Not applicable.
